# A machine learning approach using conditional normalizing flow to address extreme class imbalance problems in personal health records

**DOI:** 10.1186/s13040-024-00366-0

**Published:** 2024-05-25

**Authors:** Yeongmin Kim, Wongyung Choi, Woojeong Choi, Grace Ko, Seonggyun Han, Hwan-Cheol Kim, Dokyoon Kim, Dong-gi Lee, Dong Wook Shin, Younghee Lee

**Affiliations:** 1 School of Computing, KAIST, Daejeon, Republic of Korea; 2 College of Veterinary Medicine and Research Institute for Veterinary Science, Seoul National University, Seoul, Republic of Korea; 3 Department of Chemistry, Seoul National University, Seoul, Republic of Korea; 4 Department of Computer Science, Georgetown University, Washington, D.C USA; 5 Department of Psychiatry & Huntsman Mental Health Institute, University of Utah School of Medicine, Salt Lake City, UT USA; 6 Department of Occupational and Environmental Medicine, College of Medicine, Inha University, Incheon, Republic of Korea; 7 Department of Biostatistcs, Epidemiology and Informatics, Perelman School of Medicine, University of Pennsylvania, Philadelphia, PA USA; 8 Department of Clinical Research Design and Evaluation & Department of Digital Health, Samsung Advanced Institute for Health Science and Technology (SAIHST), Sungkyunkwan University, Seoul, Republic of Korea; 9 Department of Family Medicine & Supportive Care Center, Samsung Medical Center, Sungkyunkwan University School of Medicine, Seoul, Republic of Korea

**Keywords:** Personal health record, Class imbalance, Machine learning, Conditional normalizing flow

## Abstract

**Background:**

Supervised machine learning models have been widely used to predict and get insight into diseases by classifying patients based on personal health records. However, a class imbalance is an obstacle that disrupts the training of the models. In this study, we aimed to address class imbalance with a conditional normalizing flow model, one of the deep-learning-based semi-supervised models for anomaly detection. It is the first introduction of the normalizing flow algorithm for tabular biomedical data.

**Methods:**

We collected personal health records from South Korean citizens (*n* = 706), featuring genetic data obtained from direct-to-customer service (microarray chip), medical health check-ups, and lifestyle log data. Based on the health check-up data, six chronic diseases were labeled (obesity, diabetes, hypertriglyceridemia, dyslipidemia, liver dysfunction, and hypertension). After preprocessing, supervised classification models and semi-supervised anomaly detection models, including conditional normalizing flow, were evaluated for the classification of diabetes, which had extreme target imbalance (about 2%), based on AUROC and AUPRC. In addition, we evaluated their performance under the assumption of insufficient collection for patients with other chronic diseases by undersampling disease-affected samples.

**Results:**

While LightGBM (the best-performing model among supervised classification models) showed AUPRC 0.16 and AUROC 0.82, conditional normalizing flow achieved AUPRC 0.34 and AUROC 0.83 during fifty evaluations of the classification of diabetes, whose base rate was very low, at 0.02. Moreover, conditional normalizing flow performed better than the supervised model under a few disease-affected data numbers for the other five chronic diseases – obesity, hypertriglyceridemia, dyslipidemia, liver dysfunction, and hypertension. For example, while LightGBM performed AUPRC 0.20 and AUROC 0.75, conditional normalizing flow showed AUPRC 0.30 and AUROC 0.74 when predicting obesity, while undersampling disease-affected samples (positive undersampling) lowered the base rate to 0.02.

**Conclusions:**

Our research suggests the utility of conditional normalizing flow, particularly when the available cases are limited, for predicting chronic diseases using personal health records. This approach offers an effective solution to deal with sparse data and extreme class imbalances commonly encountered in the biomedical context.

**Supplementary Information:**

The online version contains supplementary material available at 10.1186/s13040-024-00366-0.

## Background

Personal health records (PHRs) are a comprehensive dataset that captures an individual’s health status, including medical, genetic, and lifestyle data [1]. In a conventional setup, the PHR typically encompasses data from hospital records, such as physician visits, treatment histories, test results, and prescription records [2]. However, the scope of the PHR has been expanding to incorporate genetic information like genomic sequences and life-log data [3, 4], which may include elements such as daily diet records, exercise routines, and sleep patterns. While most medical data originate from hospital environments, a significant portion of PHR data is now recorded outside hospitals or organizations, especially life-log data [5]. This data predominantly comes from healthy individuals or the general population rather than patients in a healthcare setting [6, 7].

Preventive medicine primarily focuses on health maintenance and disease prevention, especially for chronic conditions, often resulting from a complex interplay between genetic predispositions and lifestyle factors [8]. The robust and diversified data from PHRs allow for a more in-depth analysis of these factors and their interactions, offering unprecedented opportunities for early detection, risk factor identification, and disease prevention.

Detection of phenotypes or disease-specific patterns within PHRs is widely carried out using machine learning methods [1], especially classification models such as random forest [9], support vector machine [10], and light gradient boosting machine (LGBM) [11], in terms of disease prediction, diagnosis, and risk factor detection. In particular, disease development is often caused by a complex interplay of genetic and non-genetic factors such as lifestyle. Machine learning has many advantages in dealing with this complex system.

Class imbalance is an issue when the total number of samples in one class is far less than the respective totals in other classes [12]. This discrepancy can hinder the training of supervised machine learning models, which typically function under the assumption of evenly distributed classes [13]. It is especially problematic in predicting diseases since the number of people with the disease is often much smaller than those without it. Especially in the general population, the proportion of people with a particular disease will likely be much lower than in a hospital setting since most individuals are healthy. This creates an extreme class imbalance with a significantly larger ‘disease-unaffected’ class and a relatively more minor ‘disease-affected’ class. Such an imbalance presents a significant challenge for traditional machine learning approaches in predicting diseases from PHRs [14].

Despite this being a well-known significant problem, the extreme imbalance in personal health records of general diseases remains unsolved [8]. To address this issue, we introduced a semi-supervised anomaly detection model that utilizes only the majority class (i.e., non-disease) as the training set and then predicts whether a test data point is an anomaly (i.e., a patient).

Semi-supervised anomaly detection models based on normalizing flow have recently garnered attention due to their success in managing industrial anomalies [15]. Despite their promising potential, applications of normalizing flow for anomaly detection in biology and translational research remain unexplored. Our work aims to bridge this gap and develop more effective models for biomedical research, especially within the context of class imbalances typical of general population PHRs.

The goal of a normalizing flow model is to represent the vector *x* sampled from a given probability distribution as *T* (*u*) where *u* is sampled from a base probability distribution, which is usually a multivariate normal distribution. In particular, let *T* be a diffeomorphism, which means it has an inverse map ${T}^{-1}$, and both *T* and ${T}^{-1}$ are differentiable. Through invertible *T*, the space of the base distribution is skewed to create the given distribution. Since the ratio for hypervolume expansion is expressed as the absolute Jacobian determinant locally, the magnification in probability through *T* is expressed as an inverse of the absolute Jacobian determinant of *T* [16]. For efficient calculation, *T* is represented by the layer composition, which is designed so that the inverse mapping and Jacobian determinant are easily calculated.

Normalizing flow models are widely used for image generation and density estimation [17] because sampling from the multivariate normal distribution is identical to the generation of images through *T*. They have also applied for semi-supervised anomaly detection tasks, as with DifferNet [18]. In this approach, the model is exclusively trained with only “normal” data and maps these data points onto a standard normal distribution. The test data then contains both normal and anomaly data. Outputs from normal data are expected to have a high likelihood of fitting the normal distribution. Conversely, outputs derived from anomaly data are expected to show lower likelihoods to the normal distribution. This distinction aids in effectively discerning between normal and anomalous data points.

The conditional normalizing flow (CNF) has been introduced to deal with the conditional distribution. The CNF architecture ensures that each layer is transformed according to the conditional vector, which in turn allows for the accurate representation of data variations based on distinct conditions [19]. One of the notable applications of CNF is CFLOW-AD, where the CNF utilized conditional vectors derived from the two-dimensional positional encoding of industrial images for accurate anomaly localization [15]. Such models show the adaptability of CNFs in handling varied datasets.

In this study, we seek to apply a conditional normalizing flow model to resolve the extreme class imbalance problems present in PHRs.

## Materials and methods

### Data collection and incorporation

We recruited data from 706 South Korean citizens aged 19 to 59 years, 315 males and 391 females, in which there is no specific focus on disease as a random sampling of ordinary individuals was designed. The average age of all participants was 37.8 years old, with men averaging 40.4 and women 35.7 years old. Demographic details, namely the age and gender distributions, are presented in Table 1.

**Table 1:** Age and gender distribution of participants

Age group	Total	Male	Female
19	1	0	1
20–29	172	45	127
30–39	228	98	130
40–49	193	104	89
50–59	112	68	44
**Total**	706	315	391

Model features were composed of genetic, lifelog, and medical check-up data. Genetic data was collected via direct-to-customer (DTC) service (microarray chip, gen2me product from Eone-Diagnomics Genome Center). Polygenic risk scores (PRS) for 63 traits, such as cholesterol level and risk of conditions like alopecia, were provided in the DTC results. Each trait is categorized into five discernible stages. For example, the genetic probability of excessive blood glucose concentration was expressed as low, somewhat low, medium, somewhat high, and high. Non-genetic data, including medical health check-ups and lifestyle log data, were collected through a survey and a wearable device (Samsung Galaxy Fit v1, v2). The survey covered aspects of lifestyle such as smoking, drinking, and food intake tendency. Finally, the smart band recorded information, including heart rate, number of steps, and exercise for 30 days. The medical check-ups provided information such as height, weight, blood glucose level, and blood pressure.

After integrating the various data, the total number of features was 229, comprised of 63 genetic, 21 medical check-up, and 145 life-log features. Among the life-log features, 136 variables were derived from surveys and nine from smart band data. A complete list of the 229 features is shown in Supplementary Table S1.

### Target definition

**Fig. 1:**
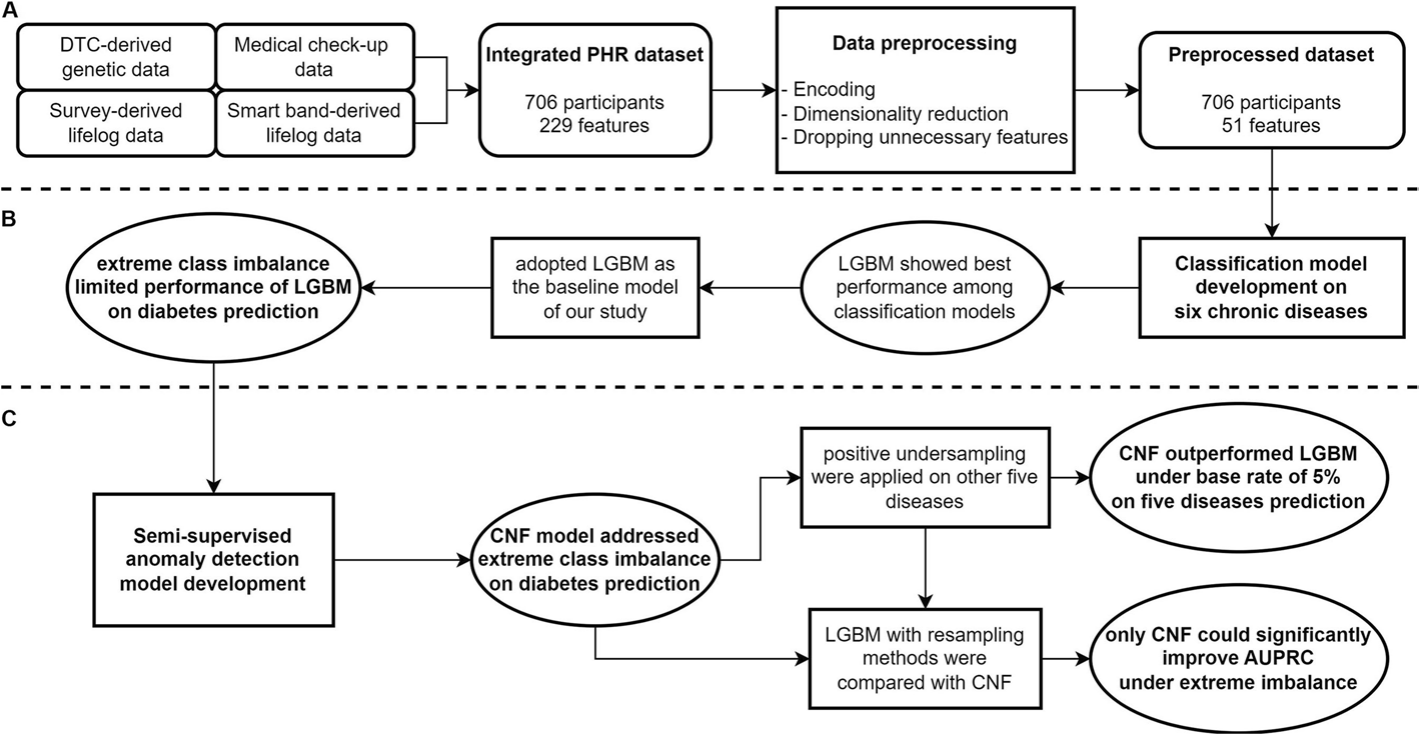
Overall research workflow. **A** Integrated version of four datasets from 706 participants is composed of 229 features (Supplementary Table S1). The integrated PHR dataset was preprocessed, ending the remaining 51 features (Supplementary Table S3). **B** Four classification algorithms – SVM, RF, LGBM, and XGBoost were used to predict six chronic diseases (obesity, diabetes, hypertriglyceridemia, dyslipidemia, liver dysfunction, and hypertension) occurrence. Extreme class imbalance limited the performance of LGBM, a baseline model, in predicting diabetes. **C** We then trained semi-supervised anomaly detection models – 1C-SVM, ISF, GMM, and CNF—to solve the performance limitation issue caused by extreme class imbalance. We also tested the models in various extreme imbalance cases and compared with the resampling methods commonly used in imbalance situations

Figure 1 provides a schematic representation of our overall workflow. We first aimed to model machine learning algorithms for predicting six chronic diseases: obesity, diabetes, hypertriglyceridemia, dyslipidemia, liver dysfunction, and hypertension. Participant disease status was determined according to biomarkers extracted from their medical check-up data. We assigned disease status as a binary class, categorizing them as either ‘affected’ or ‘unaffected’ for each participant based on the simple categorical thresholds listed in Supplementary Table S2. Biomarkers corresponding to each specific disease were excluded while fitting machine learning models. Any data points with missing values in the target variables were excluded from the analysis to ensure robustness and accuracy.

### Data preprocessing

Features exhibiting right-skewed distributions, such as AST, ALT, neutral fat, and step count per day, underwent log-transformation to better approximate normal distributions. Because an excessively large number of features compared to the sample size may hinder the models’ accuracy and interpretability, we filtered out inconsiderable features [20, 21]. Certain features were dropped due to sparseness or lack of clinical relevance. Specifically, features such as house floor material, house room number, and road traffic experience were deemed clinically irrelevant to the chronic diseases under investigation.

The data were partitioned into training and test sets at a ratio of 8 to 2, respectively, for standardized measurement of generalized model performance [22]. Missing values were imputed with an iterative imputer and standardized with a robust scaler [23]. To address multicollinearity among correlated features (e.g., smoking duration and daily cigarette consumption, various secondary smoking experience features), we applied principal component analysis (PCA) to relevant feature subsets.

To prevent data leakage, all preprocessing techniques, including missing value imputation, scaling, and PCA, were fitted to the training set. The test set was then transformed using the parameters learned from the training set. After preprocessing, 51 features remained for modeling. The complete list of these 51 features and detailed preprocessing steps are provided in Supplementary Table S3.

### Supervised machine learning model fitting for classification as a baseline performance

We trained four supervised classification machine learning models in preparation for this study, including well-recognized algorithms like support vector machine (SVM), light gradient boosting machine (LGBM), random forest, and extreme gradient boosting. Class weights were used to calibrate imbalance labels. To optimize the models, hyperparameters were tuned via a five-fold cross-validation in the training set.

Meanwhile, tree-based algorithms are relatively powerful at capturing irregular patterns, which makes them advantageous for tabular datasets [24], and LGBM is a well-known model in this matter. Therefore, we adopted LGBM as our baseline model for our study. Most of all, LGBM has been designed to be more efficient in terms of computational speed, memory usage, generalized outstanding performance, and efficiency, and it is widely used in various research domains [25].

### Machine learning models for semi-supervised anomaly detection: one class support vector machine (1C-SVM), isolation forest (ISF), and gaussian mixture model (GMM)

We utilized 1C-SVM, ISF, and GMM modeling approaches, training both models exclusively on data from unaffected individuals.

Unlike a typical binary SVM, 1C-SVM creates boundaries with only normal classes: that is, it additionally assumes that the origin of the feature space (where the kernel function is mapping to) is an anomaly. Thus, a hyperplane separates normal points in the feature space from the origin. Ultimately, 1C-SVM aims to find the plane farthest from the origin, and adds a relaxed form of constraint into the loss to prevent misjudgment [26].

Isolation forest, one of the representative semi-supervised algorithms, constructs isolation trees by random selection of a feature and a split value in subsamples of the dataset. Anomalies are identified by their shorter paths in the tree, as they are isolated more quickly than the densely located normal samples [27].

Gaussian mixture model (GMM), on the other hand, is a well-known algorithm designed to approximate any arbitrary distribution as a mixture of multiple gaussian distributions, represented as:

$$f\left(x\left|\theta\right.\right)=\Sigma w_if_i\left(x\right),f_i\left(x\right)\sim N_m\left(\mu_i,\;\Sigma_i\right),\theta=\left(W_i,\;\mu_i,\;\Sigma_i\right)$$

for $\sum {w}_{i}=1, {w}_{i}\ge 0$ [28]. The likelihood $f(x|\theta )$ of the test set can be utilized for inference.

After subjecting the models to a preprocessing routine similar to that used for prior supervised classification models, hyperparameters were ascertained through evaluation on a validation set.

### Benchmark with state-of-the-art deep learning models: TabNet, FT-Transformer, and GOAD

In addition to the above mentioned models, we further benchmarked our data with three state-of-the-art deep learning models designed explicitly for tabular data: TabNet, FT-Transformer, and GOAD.

TabNet [29] is a deep learning architecture that utilizes sequential attention to choose which features to reason from at each decision step, enabling interpretability and better learning as the network can learn to focus on the most salient features. The model consists of a feature transformer, an attentive transformer, and a classifier. The feature transformer processes the raw input features, the attentive transformer employs a sparsemax function to select the most relevant features, and the output is aggregated from the processed features at each step to make the final prediction.

FT-Transformer [30] is an adaptation of the Transformer architecture [31] for tabular data. It employs a feature tokenizer to convert the input features into a sequence of embeddings, which are then processed by standard transformer layers. The [CLS] token embedding from the Transformer output is used for final prediction. The model also incorporates normalization techniques and GLU activations to improve optimization stability and performance.

GOAD [32] is a deep learning approach for anomaly detection that achieves the state-of-the-art performance in broad types of data, including tabular datasets. GOAD trains a classifier to predict the applied transformation on both normal and transformed data points. The approach assumes that the transformed normal samples are likely to fall in the corresponding subspace modeled by the network, while anomalies would deviate from the learned manifold. The classification probability is used to compute an anomaly score.

### Conditional normalizing flow model

#### Training loss

To tackle the extreme class imbalances within our dataset, particularly in the distinction between affected and unaffected individuals, we introduced the CNF model.

Given an invertible mapping between a multivariate normal distribution (*Z*) and a more intricate distribution (*X*), PHRs from unaffected individuals are used to construct the maximum likelihood:

$$\text{log}\ p_X\left(x;\theta,\psi\right) = \text{log}\ p_Z\left(f_\theta\left(x\right);\psi\right)+\log \left|\det\left(\frac{\partial{\text{f}}_{\theta}\left(\text{x}\right)}{\partial\text{x}}\right)\right|$$

where $\theta$ and $\psi$ represent the parameters associated with the model and the base distribution (*Z*), respectively. Training loss is captured by the negative log-likelihood, as is done for general normalizing flow models. This formulation offers a strategic approach to address the class imbalance issue [16].

#### Conditional affine transformation

To account for the variations in PHR distributions influenced by age and gender, we incorporated a conditional affine transformation, which takes conditions into account sufficiently. Specifically, since age and gender have significant influences on health conditions [33], we set these two features as a conditional vector $c\in {\mathbb{R}}^{2}$ and thus excluded from the input vector $x\in {\mathbb{R}}^{50}$.

A conditional affine transformation $\left(\text{x}|\text{c}\right)\mapsto \text{u}$ operates as per the following [34]:

$$\text{a},\text{b}=\text{SPLIT}\left(\mathrm s\left(\mathrm c\right)\right)$$

$$\text{a}^{\prime}=\upalpha\uppsi\left(\text{a}\right)$$

$$u=\text{exp}\left(\mathrm a'\right)\odot x+b$$

  

Here, the function $s:{\mathbb{R}}^{2}\to {\mathbb{R}}^{48}$ signifies a neural network architecture, which is constructed with a sequence of a linear layer, a GeLU activation function, followed by another linear layer. The hyperparameter α is a constant, while Ψ denotes an activation function. The symbol ⊙ denotes the Hadamard product, an element-wise multiplication. The SPLIT function divides $s(c)\in {\mathbb{R}}^{48}$ into two distinct vectors, $a, b\in {\mathbb{R}}^{24}$. Simply, the coefficients of affine transformation, $a$ and $b$, are induced by two independent neural networks.

The coefficients of affine transformation are derived from the conditional vector. Finally, the affine transformation is performed on the input x. This configuration enables the data to be modified based on their age and gender, owing to this unique structure.

#### General incompressible-flow networks (GIN) coupling blocks

Coupling block, one of computationally efficient bijective transformation, is a key structure in our conditional normalizing model in terms of provision of representability. In particular, we stacked double conditional GIN coupling layers to make coupling layers preserve volume, based on the expanded theory of nonlinear independent component analysis (ICA) for problems with unknown intrinsic dimensions [35]. A global offset ${t}_{\text{global}}$ and permutation with $R$ were also applied for better representability. The specific process can be expressed as:

$$R{\text{Coupling}}\left(x,c\right)+{t}_{\text{global}}$$

Within the confines of the coupling layer, $c$ persists as the conditional vector. We additionally split $x\in {\mathbb{R}}^{48}$ into two subsets: ${x}_{1}\in {\mathbb{R}}^{24}$ and ${x}_{2}\in {\mathbb{R}}^{24}$. Subsequently, the transformation proceeds as ${\text{CONCAT}}\left({u}_{1},{u}_{2}\right)$, where:

$$u_1=x_1\odot exp(\tanh\left(s\left(\mathrm{CONCAT}\left(x_2,\;c\right)\right)\right)+t\left(\mathrm{CONCAT}\left(x_2,\;c\right)\right)$$

$${u}_{2}={x}_{2}$$

Here, ${\text{CONCAT}}$ represents vector concatenation. The functions $s$ and $t$ denote subnetworks, which are architecturally composed of a linear layer, followed by a ReLU activation, and another linear layer. The unique was proposed to simplify the calculation of the Jacobian determinant [17]. The normalizing flow implementation from FrEIA was utilized in our study [36].

#### Strategy for optimizing CNF model training

To train the above-described models, we adopted the Adam optimizer [37] and the Cosine Annealing Warmup Restart scheduler [38]. These were chosen to ensure efficient and effective model training. Additionally, to further enhance the optimization and representability of the model, we introduced AltUB, which periodically trains the parameters of the base distribution [39].

#### Inference

The evaluation of anomalies was measured using a scoring system defined as:

$$-\text{exp}\left(\text{-}\frac{z^T z}2\right)$$

which is negatively proportional to the likelihood of $z$. A lower likelihood implies that the given test point bears a greater resemblance to an anomaly. Outputs from disease samples are expected to have a lower likelihood and thus, a higher anomaly score.

**Fig. 2:**
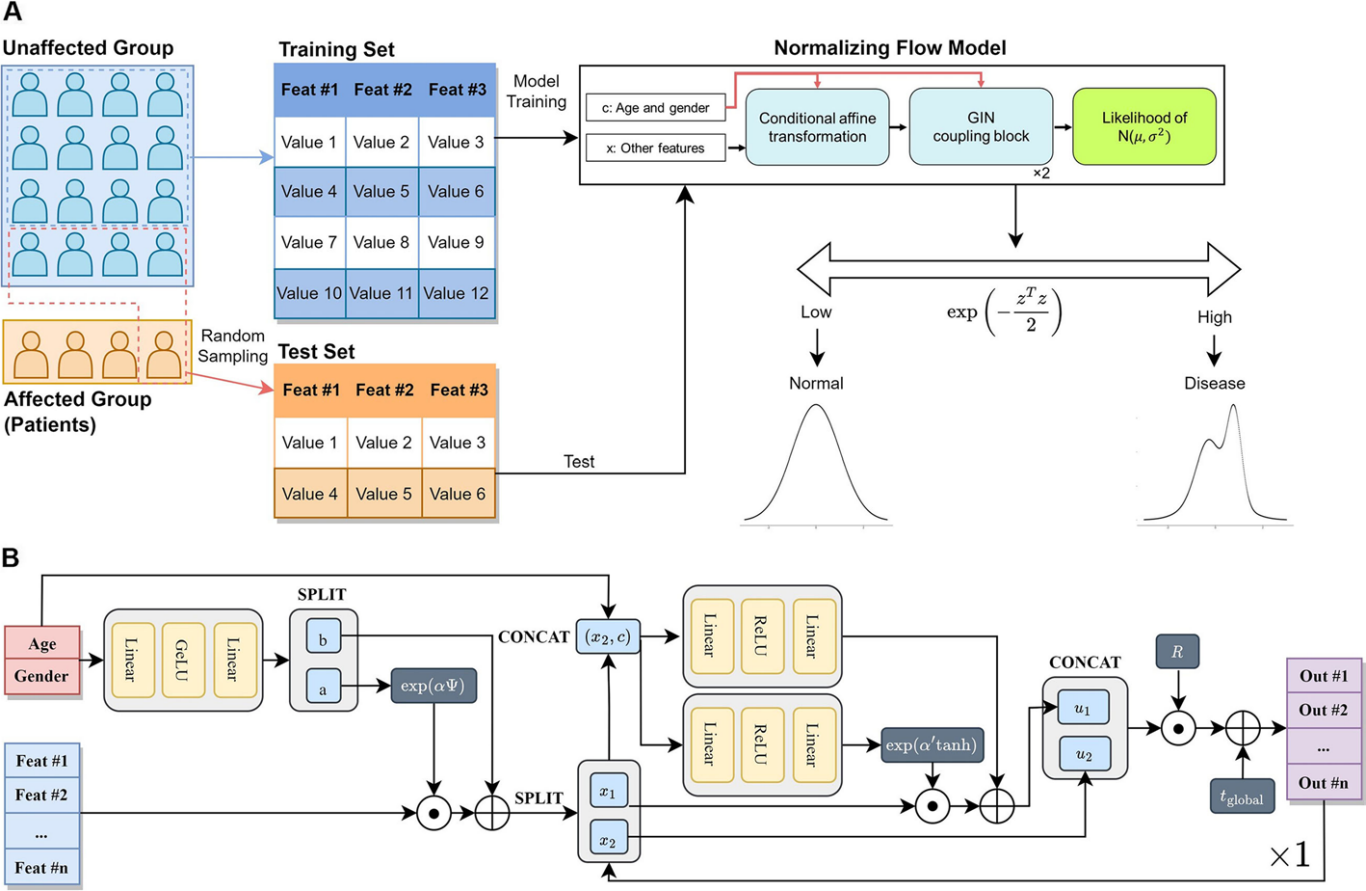
Architecture of the CNF model. **A** Within the framework of semi-supervised anomaly detection models, only the unaffected data, which represents the controls, is employed during the training phase. The test set encompasses both unaffected and affected data so as to assess model performance. To conserve base proportions, test sets were determined through random sampling. In the conditional normalizing flow model for anomaly detection, the unaffected data are mapped into a multivariate normal distribution (m. n. d). The affected data, representing the patients, undergoes a transformation resulting in a non-standard distribution. The difference between these distributions is quantified using the metric $\text{exp}\left(-\frac{{z}^{T}z}{2}\right)$, which is negatively proportional to the likelihood of m. n. d. Here, $z$ symbolizes the resultant output of the CNF model. **B** The comprehensive pipeline of the CNF model for semi-supervised anomaly detection. Notably, age and gender are taken as a bi-dimensional conditional vector, underscoring their significance in the model’s architecture

To ensure fair and robust performance comparisons between models, we stratified evaluation steps: the proposed CNF model was evaluated with multiple sampling cases that conserved the base rate. First, only unaffected samples were divided into a training set and a test set using 8:2 splitting. Then, affected samples were partitioned into five subsets. Each of these subsets was merged with unaffected data. This step ensured that each of the test datasets maintained base rates analogous to that of the original dataset. Subsequent evaluations were conducted across these datasets, with performance metrics averaged to provide a comprehensive assessment of the model's robustness and adaptability. The full process and architecture of the proposed CNF model is graphically presented in Fig. 2.

### Metrics

Prediction models were evaluated using the area under the receiver operating characteristics curve (AUROC) and also the area under the precision-recall curve (AUPRC). The baseline AUROC value (the performance of a random estimator) is always 0.5, whereas the base value of AUPRC is the proportion of positive data. A key difference between AUROC and AUPRC is that the latter prioritizes how well the model handles positive samples, while the former considers handling positive and negative samples equally. AUPRC is thus regarded as the more appropriate metric when false negatives are more consequential than false positives, such as in the prediction of chronic diseases [40]. Therefore, we considered AUPRC as the primary metric in our research.

## Results

### Data characteristics

The total number of participants was 706 individuals, with 44.6% males and 55.4% female. Their ages ranged from 19 to 59 years. Participant disease status was determined using the simple categories as described in Materials and methods (Supplementary Table S2). We identified the following disease prevalence among the participants: 230 (32.6%) as having obesity, 13 (1.8%) diabetes, 58 (8.2%) hypertriglyceridemia, 56 (7.9%) dyslipidemia, 81 (11.4%) liver dysfunction, and 242 (34.3%) hypertension. As shown above, diabetes prevalence status was extremely imbalanced (1.8% of the population were diabetes-affected).

### Performance of machine learning models on predicting chronic disease occurrence

**Fig. 3:**
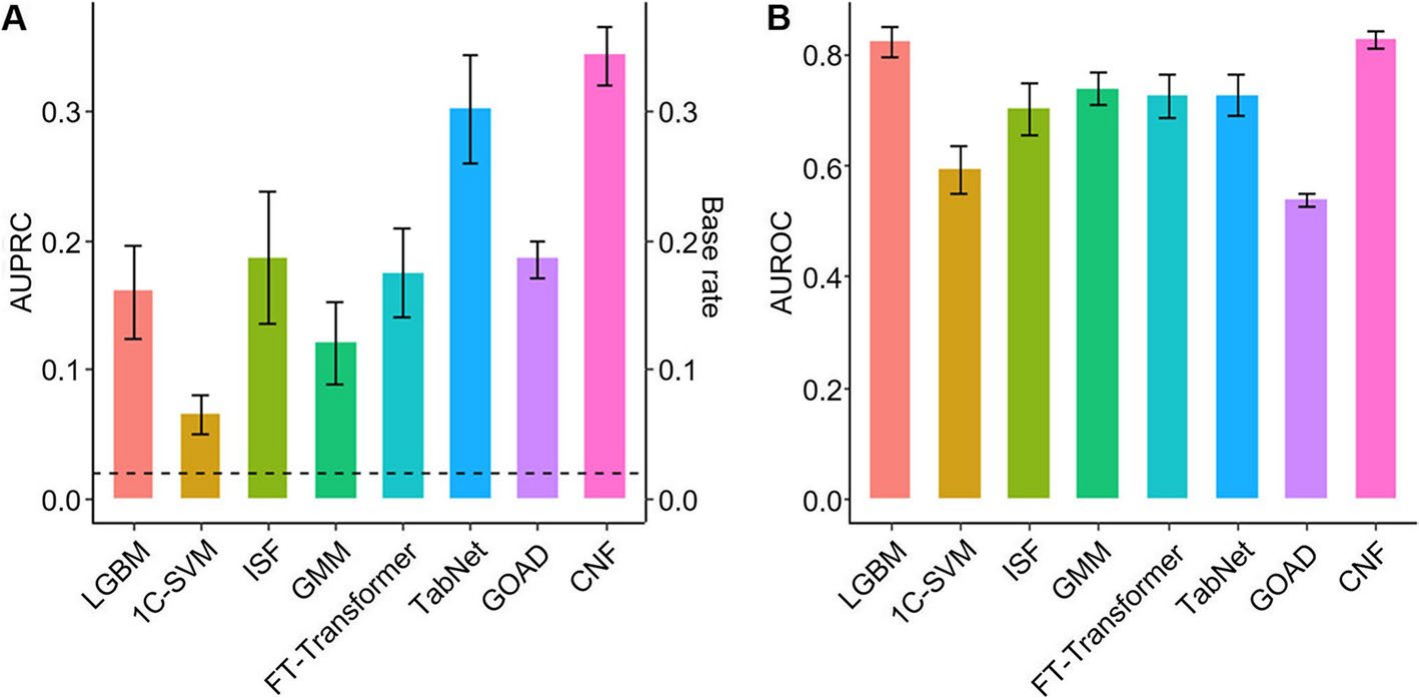
Performance of eight models in predicting diabetes. All 95% confidence intervals were determined through 50 repetitions. **A** AUPRC of the models. The base rate (= 0.02) is indicated as a dashed line. The conditional normalizing flow (CNF) model achieved an AUPRC of 0.34, which was significantly high and stable. **B** AUROC of the models. LGBM and CNF achieved high values compared to other algorithms at about 0.83

LGBM emerged as the best classification model for the prediction of the six chronic diseases regarding overall AUROC, AUPRC, and F1 score (Supplementary Fig. S1). The average AUROC and AUPRC values, accompanied by their respective 95% confidence intervals derived from LGBM, are detailed in Table 2. Due to the extreme class imbalance for diabetes, the corresponding AUPRC value was low (0.16) associated with diabetes prediction. Although this value may seem commendable given the foundational base ratio (0.02), it underscores potential enhancement in the model’s performance. Motivated by this observation, we applied a semi-supervised anomaly detection models (1C-SVM, ISF, and GMM) with CNF specifically for diabetes, as illustrated in Fig. 3. Additionally, the state-of-the-art models, TabNet, FT-Transformer, and GOAD were evaluated. As a result, CNF achieved the highest AUPRC (0.34) with comparable AUROC (0.83). Supplementary Fig. S2 summarizes the performance of LGBM and CNF on every investigated disease. A comparison between LGBM and CNF revealed that LGBM achieved better AUPRC values for the other five chronic diseases, but for diabetes, which exhibited an extremely low prevalence, CNF outperformed LGBM by achieving a higher AUPRC. In short, the CNF could cope with class imbalance better than LGBM and the other models for classification or semi-supervised anomaly detection.

**Table 2:** AUROC and AUPRC of LGBM in chronic diseases

LGBM	AUROC	AUPRC	Base rate
Obesity	0.82 ± 0.01	0.73 ± 0.02	0.33
Diabetes	0.82 ± 0.03	0.16 ± 0.04	0.02
Hypertriglyceridemia	0.87 ± 0.01	0.51 ± 0.02	0.08
Dyslipidemia	0.89 ± 0.01	0.55 ± 0.03	0.08
Liver dysfunction	0.92 ± 0.01	0.65 ± 0.03	0.11
Hypertension	0.72 ± 0.01	0.56 ± 0.02	0.34

### Evaluation of model performance in further extreme imbalance cases

**Fig. 4:**
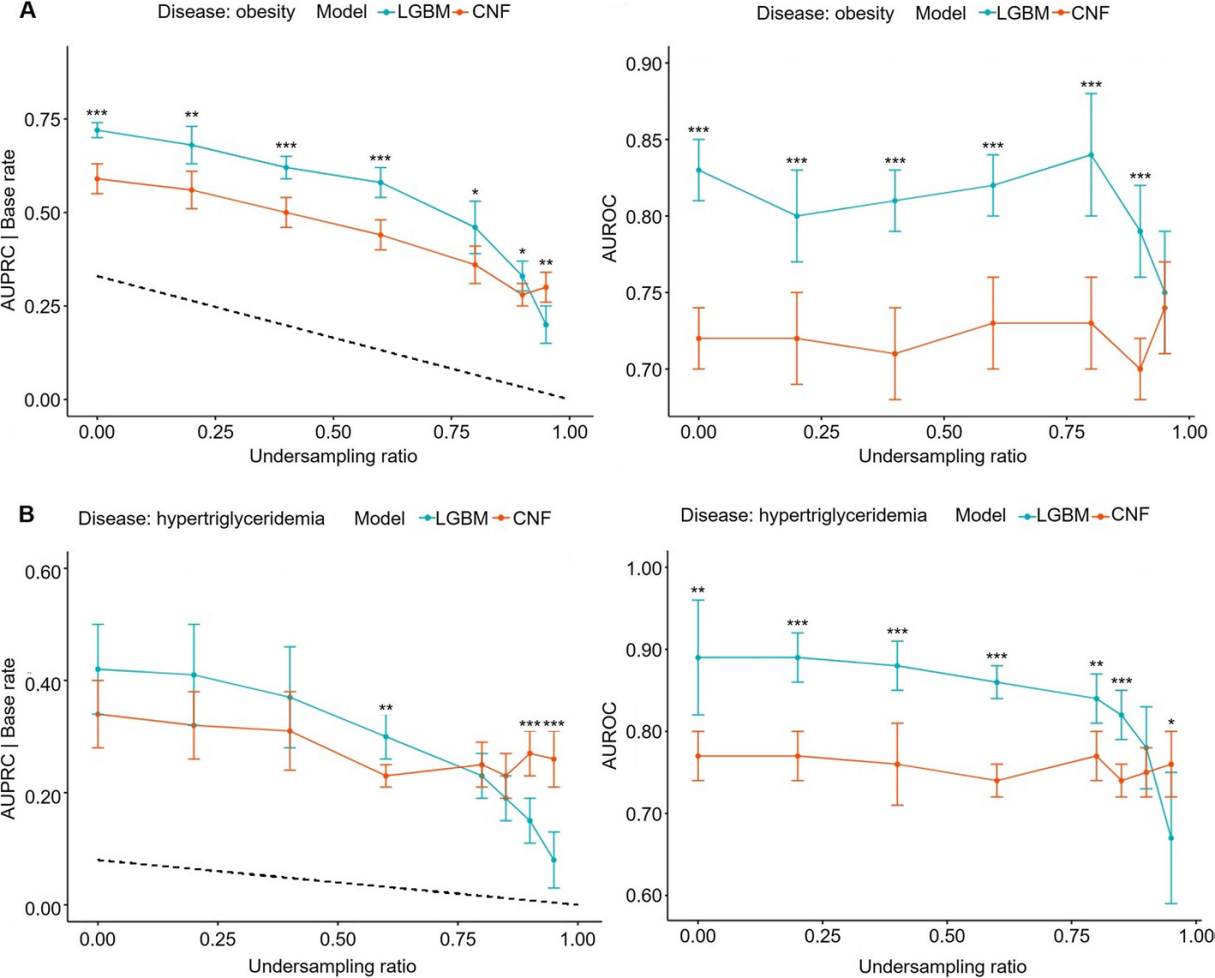
The relationship between positive undersampling ratio and performance of LGBM (blue) and conditional normalizing flow (CNF, red) models on two chronic diseases: (**A**) obesity, (**B**) hypertriglyceridemia. The dashed line indicates the actual base rate after adjustment of the number of positive samples. The left and right panels represent AUPRC and AUROC, respectively. The AUPRC of LGBM dropped dramatically with extremely low base rates. (**A**, **B**) Statistical analysis by Welch's t-test, *p < 0.05, **p < 0.01, ***p < 0.001

To further investigate whether our model works on which level of imbalance degree, regardless of the target disease, we systematically excluded 20% to 95% of the disease-affected samples from our dataset (positive undersampling). Then, LGBM and CNF models were evaluated with performance metrics. The results can be found in Fig. 4 and Supplementary Fig. S3. Interestingly, as the base rate gradually decreased, the AUROC values did not significantly differ between the two model types. However, in scenarios where the base rate was extremely low, the CNF model exhibited a pronounced superiority in AUPRC over the LGBM. For example, while LGBM performed AUPRC 0.20 and AUROC 0.75, CNF showed AUPRC 0.30 and AUROC 0.74 when predicting obesity in a 95% positively undersampled dataset.

### The effect of positive oversampling and negative undersampling methods

**Fig. 5:**
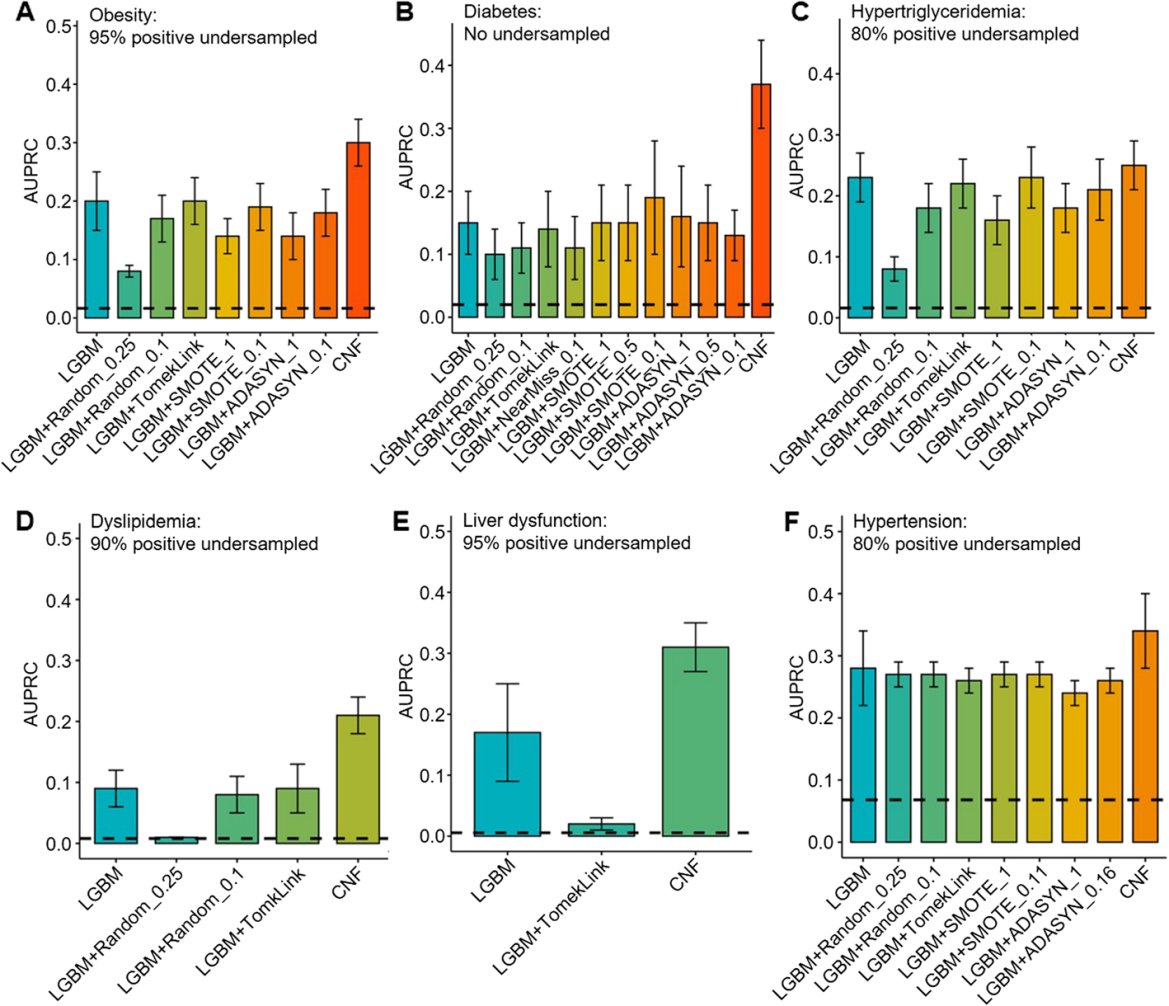
The effects of positive oversampling and negative undersampling methods on AUPRC. LGBM models were constructed with (positive) over- or (negative) undersampling methods for addressing class imbalances, namely Tomek Link, SMOTE, and ADASYN, for six chronic diseases: (**A**) Obesity: 95% undersampling, 0.02 base rate, (**B**) Diabetes: no undersampling, 0.02 base rate, (**C**) Hypertriglyceridemia: 80% undersampling, 0.02 base rate, (**D**) Dyslipidemia: 90% undersampling, 0.01 base rate, (**E**) Liver dysfunction: 95% undersampling, 0.01 base rate, and (**F**) Hypertension: 80% undersampling, 0.07 base rate. The dashed line indicates the actual base rate after adjustment of the number of positive samples. Positive undersampling was performed as needed to create a class imbalance situation. In the case of extreme class imbalance, (positive) oversampling or (negative) undersampling had little beneficial effect on the performance of LGBM-based classification models, whereas CNF showed consistently good performance

While integrating class weights offers a mechanism to manage class imbalances in LGBM, we observed a decline in model performance in situations characterized by profound imbalances, especially when base rates plummeted below 5%. Resampling techniques, which include (positive) oversampling – increases the number of minority class—and (negative) undersampling – decreases that of the majority class—have been canonically employed to rectify class imbalances. In our study, we applied resampling methods such as Tomek Link [41], SMOTE [42], and ADASYN [43] to LGBM models. These methods were particularly tested under conditions of severe class imbalance, where the AUPRC values significantly declined, as illustrated in Fig. 4 and Supplementary Fig. S3. However, none of the resampling strategies demonstrated higher AUPRC compared to the baseline (Fig. 5). In contrast, the CNF model exhibited a robust capability, outperforming the traditional resampling methods in terms of AUPRC. Furthermore, which presents AUROC comparisons, all methods yielded relatively similar AUROC values, showcasing no substantial differences (Supplementary Fig. S4). This suggests that while the CNF model offers advantages in addressing AUPRC in severe imbalanced scenarios, its performance in terms of AUROC remains consistent with other traditional methods.

## Discussion

### CNF as the best predictor in the extreme class imbalance (diabetes)

LGBM predicted most of the examined diseases well. However, the model did not successfully predict diabetes, achieving an AUPRC of 0.16, largely attributable to the significant class imbalance with a base rate of just 2%. As presented in Fig. 3, our CNF model outperformed various classification and anomaly detection models, including state-of-the-art deep learning models, and managed to double the AUPRC value of LGBM (0.34 vs. 0.16) while maintaining comparable AUROC values. Furthermore, as depicted in Fig. 4, as the base rate decreases, indicating heightened class imbalance – the CNF model becomes more pronounced in contrast to LGBM. Notably, our semi-supervised anomaly detection approach is inherently robust against overfitting issues since the models are trained solely on disease-unaffected samples and aim to identify disease-affected instances as anomalies during testing. The consistent AUROC performance across varying base rates further corroborates this robustness (Fig. 4). These results underscore the potential of CNF for tasks dominated by class imbalances, suggesting that CNF can be specialized for these tasks and merits more research in this area.

**Fig. 6:**
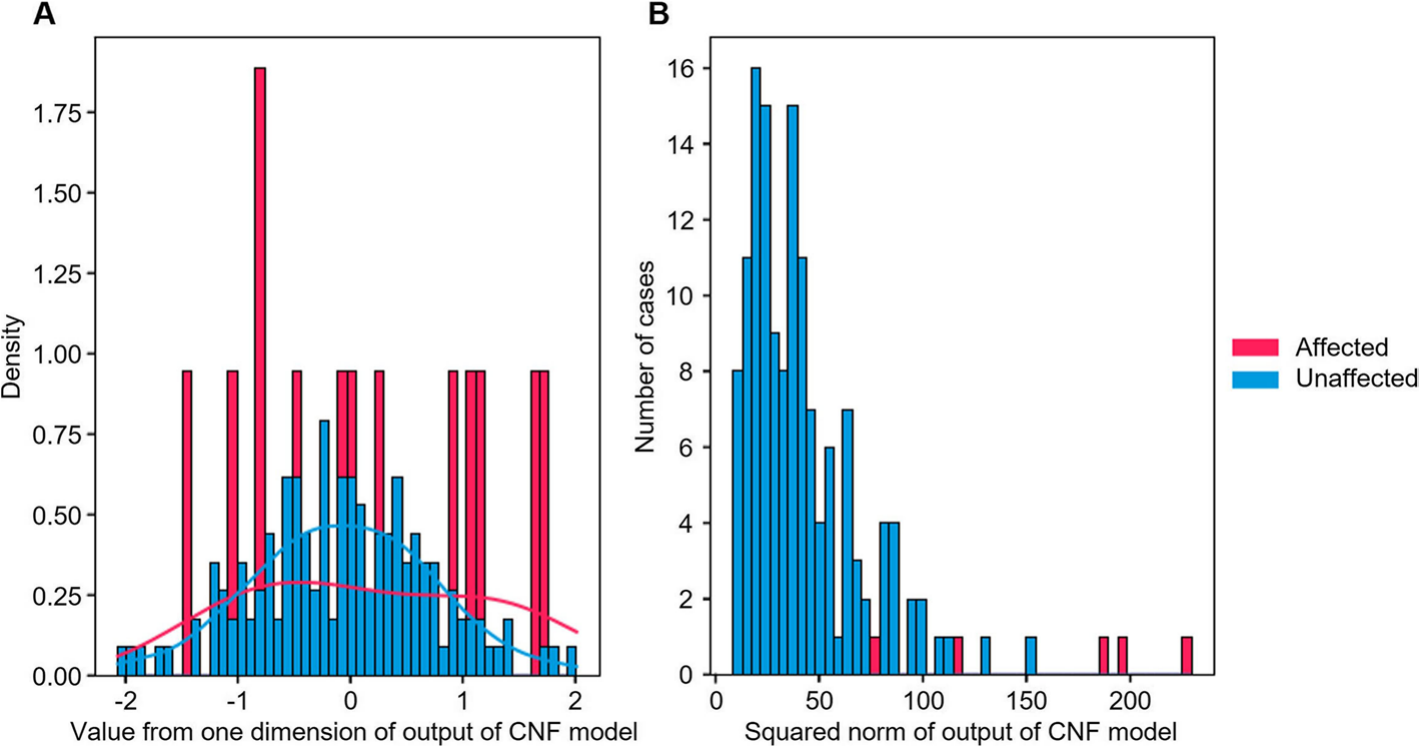
**A** The marginal distribution of outputs from applying the CNF model for diabetes to the test set. While outputs from unaffected samples follow the normal distribution, those from diabetes-affected cases do not. **B** Squared norm of output from the test set. Outputs from cases tend to be farther from the origin than controls

Figure 6(A) illustrates the transformation process to construct a normal distribution through CNF. The data from non-diabetic participants from the test dataset converges toward a normal distribution with a mean of zero. In contrast, the data from patients with diabetes were transformed into a distribution distinct from the normal, as per the previous assumption. The histogram presented in Fig. 6(B) further elucidates this conversion, where outputs from diabetic patients are observed to be farther from the origin, indicating their divergence from the standard normal distribution *N* (0, 1).

These observations suggest that while conventional classification algorithms can exhibit satisfactory performance for predicting chronic diseases when provided with a sufficient number of affected samples, they show weakness in the face of extreme class imbalances. In such situations, CNF models emerge as a more robust alternative. Since normalizing flow models have enormous expressive power, they also perform better than general semi-supervised anomaly detection models. In the case of predicting diabetes, in particular, the failure of 1C-SVM (Fig. 3) implies that the topology of diabetes is highly complex since it is susceptible to noise [44]. CNF is well-equipped to navigate and address these complexities.

### First attempt of normalizing flow on tabular PHRs

Normalizing flow has been utilized for tasks such as density estimation, data synthesis [17], and anomaly detection [18]. Although models based on conditional normalizing flow like CFLOW-AD have achieved state-of-the-art performance in diverse fields for anomaly detection, few studies applied the idea in clinical and biological domains. Within these domains, the focus of normalizing flow has usually been on density estimation and data generation, rather than anomaly detection [45–47]. Another notable point is that the majority of current and previous applications of normalizing flow concern images. For instance, the application of normalizing flow for identifying out-of-distribution was introduced for coronary artery segmentation [48].

Thus, our work, in which a CNF model is applied to predict chronic diseases from tabular PHR data, represents a pioneering effort. It marks the first attempt to apply CNF to chronic diseases such as diabetes, which seeks to improve imbalance problems from the perspective of anomaly detection.

### CNF performance according to the degree of class imbalance

The performance (especially AUPRC) of LGBM, as a supervised classification model, was always constrained by low base rates regardless of the diseases, as shown in Fig. 4. On the other hand, while CNF may exhibit suboptimal performance compared to LGBM at higher base rates, its power becomes distinctly apparent when the base rate plunges below approximately 5%. Furthermore, conventional classification models often lean on resampling strategies to mitigate the challenges posed by class imbalance. Yet, as Fig. 5 shows, these traditional methods exhibited small efficacy on our dataset. Such findings strengthen the argument for the utility of CNF, which remains effective in the face of pronounced class imbalances.

Recent works have also illuminated the potential of generative models, such as generative adversarial networks and variational autoencoders [49, 50], as solutions to class imbalance problems. While these models exhibit promise, their training processes can be intricate, and optimal performance is not always guaranteed. Consequently, the utilization of CNF is a promising and pragmatic strategy, particularly when confronted with datasets characterized by limited affected samples or extreme class imbalances.

### Necessity of the conditional vector

The conditional vector was critical, affording the model to account for age and gender-specific distributions. Supplementary Fig. S5 illustrates the associations between the gender and age, and the overall patterns of PHRs. In the broader context of medicine, it is widely recognized that these two features exert significant influence on health conditions, including the predisposition to chronic diseases [33]. Hence, the proposed model incorporated a conditional affine transformation, which maximizes the influence of age and gender during the training process. In addition, conditional coupling layers further emphasized the model’s sensitivity to the complexity of age and gender dynamics.

### Usage of AltUB

AltUB was proposed to address the parameter-shift phenomenon observed in normalizing flow models [39]. During our research, we also observed the phenomenon during modeling, indicating a potential enhancement in model performance through the incorporation of AltUB. However, the training process sometimes became unstable. Thus, the usage of AltUB can be optional based on the input dataset.

### Limitations and future works

The major limitation of our study is that the data size is small, at 706 samples. However, this study is still meaningful since the data were collected from various sources, including genetic data, medical check-ups, surveys, and wearable devices. In addition, we were able to fit machine learning algorithms, even with this small and imbalanced dataset, to improve its prediction power of chronic disease.

Our approach can be seamlessly adapted to diverse medical situations. Since many diseases have a low incidence, the challenge of class imbalance problem would play a significant role when applying canonical supervised classification algorithms. In the future, we will adopt CNF to break down the imbalance problems for rare diseases.

While the current study focused on various machine learning algorithms on PHRs for predicting chronic diseases, our further study will extend these methods to predict the risk factors associated with these diseases. In addition, we will identify critical genetic and non-genetic features among chronic diseases. Extending our current research focus will seek to enhance the ability of the CNF to accommodate a wider range of diseases by using techniques such as masking and householder permutation. Based on our approach, we expect to provide improved solutions to participants and introduce a new machine learning method for further health research.

## Conclusion

In this research, we introduced a conditional normalizing flow designed for anomaly detection, aiming to address extreme class imbalances rampant in personal health records. This study significantly improved prediction performance compared to traditional methods such as LGBM and 1C-SVM. In particular, for diabetes prediction with a base rate of 2%, conditional normalizing flow (AUPRC = 0.34) was higher than the baseline model LGBM (AUPRC = 0.16). In conclusion, the conditional normalizing flow can be a promising solution for dealing with extreme class imbalance problems on personal health records. This approach not only augments the precision of predictive modeling in the realm of medical informatics but also provides a new avenue in biomedical research.

### Supplementary Information

Supplementary Material 1.

Supplementary Material 2.

## Data Availability

The datasets generated and/or analyzed during the current study are not publicly available due to the informed consent. The code of the suggested model of the paper is available in the GitHub repository (“https://github.com/snubmi/PHR-NF”).
