# Supplementary material for: A machine learning approach using conditional normalizing flow to address extreme class imbalance problems in personal health records
Source: BioData Min. 2024 May 25;17:14. doi: 10.1186/s13040-024-00366-0 (PMC11127363; doi:10.1186/s13040-024-00366-0)
Supplement: Supplementary file 1 — Supplementary Material 1. [file 13040_2024_366_MOESM1_ESM.pdf]

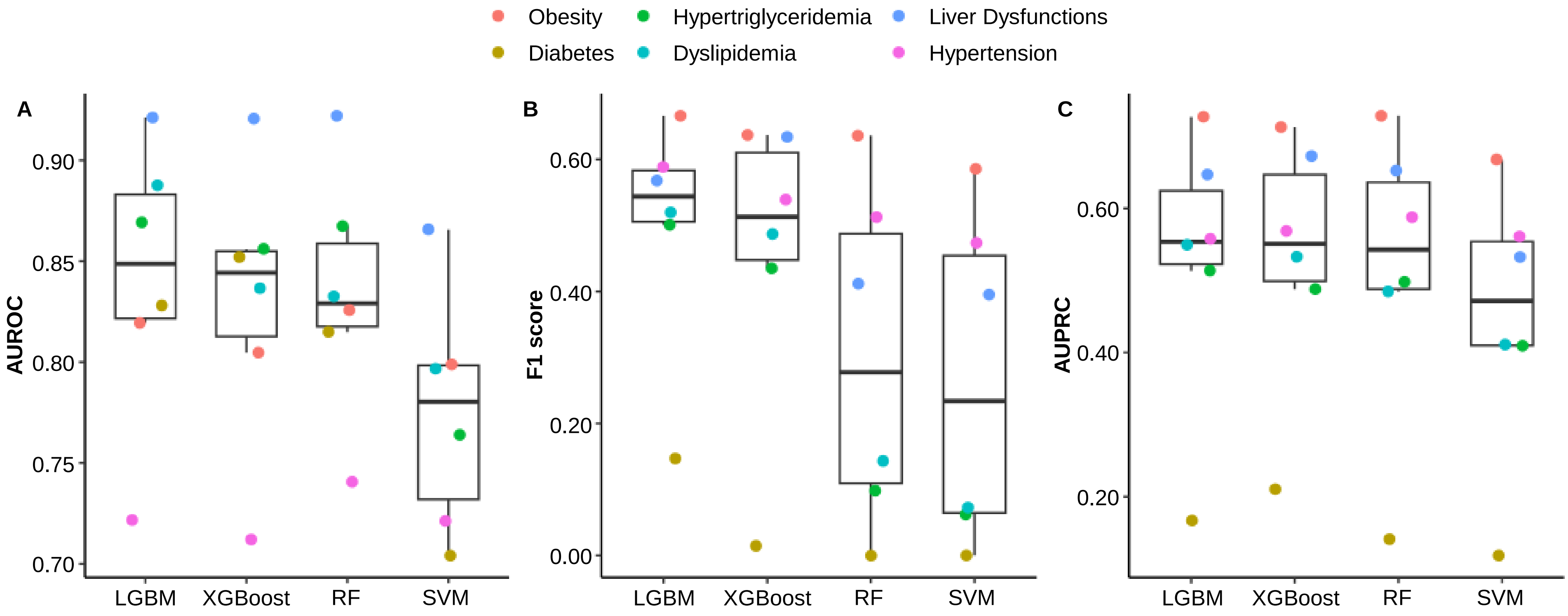

Supplementary Figure S1. Performance of representative machine learning models. Performance of models on six chronic diseases are displayed. The black line in the bar denotes the average of the metric from the six diseases. LightGBM clearly outperformed the other algorithms, in terms of (A) AUROC, (B) F1 score, and (C) AUPRC.

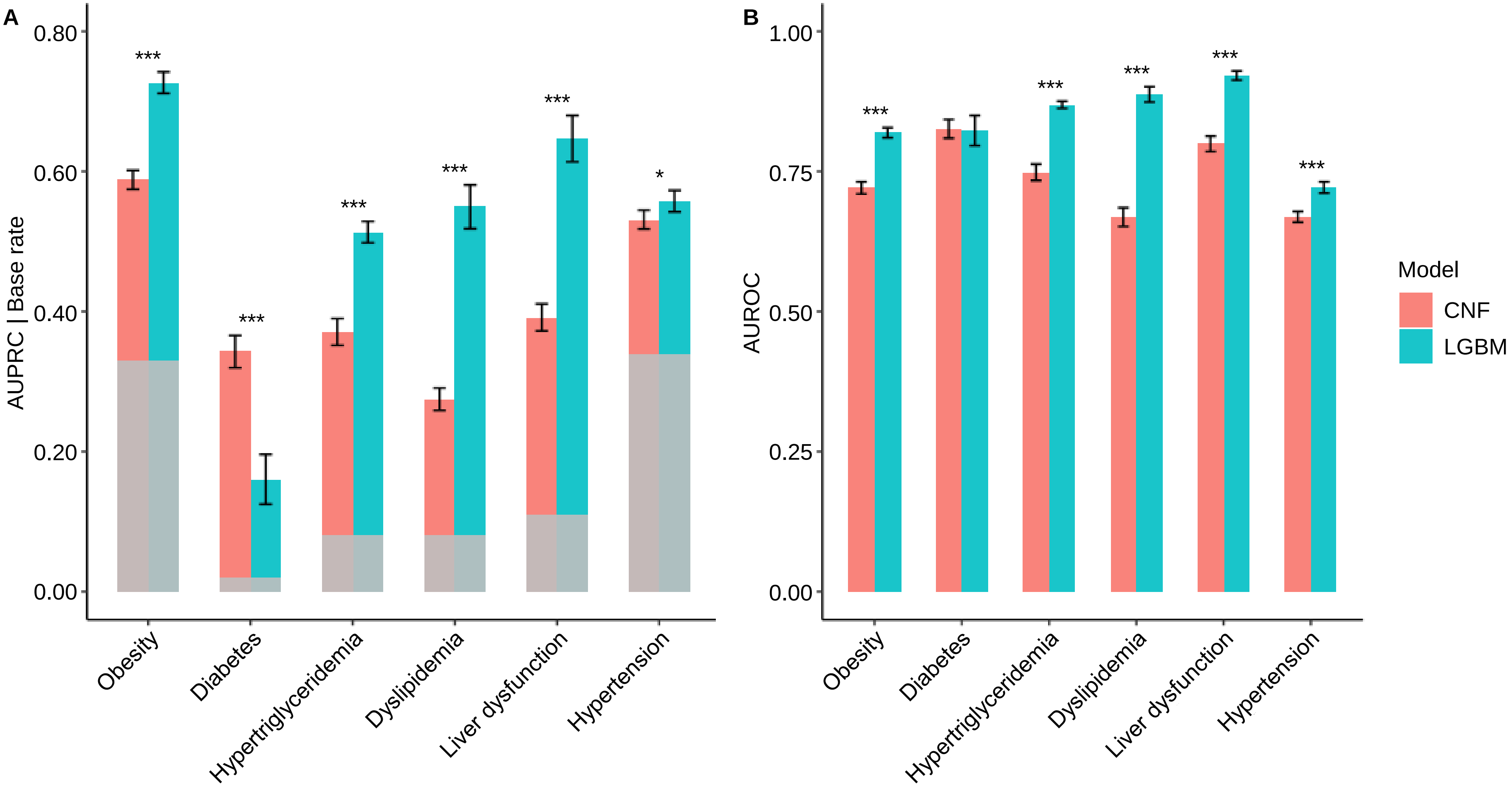

Supplementary Figure S2. Performance of LGBM and CNF on predicting six chronic diseases. All 95% confidence intervals were determined through 50 repetitions. (A) AUPRC values and associated base rates. Base rates are depicted as grey. (B) AUROC values. (A, B) Statistical analysis by Welch's t-test, *p<0.05, **p<0.01, ***p<0.001.

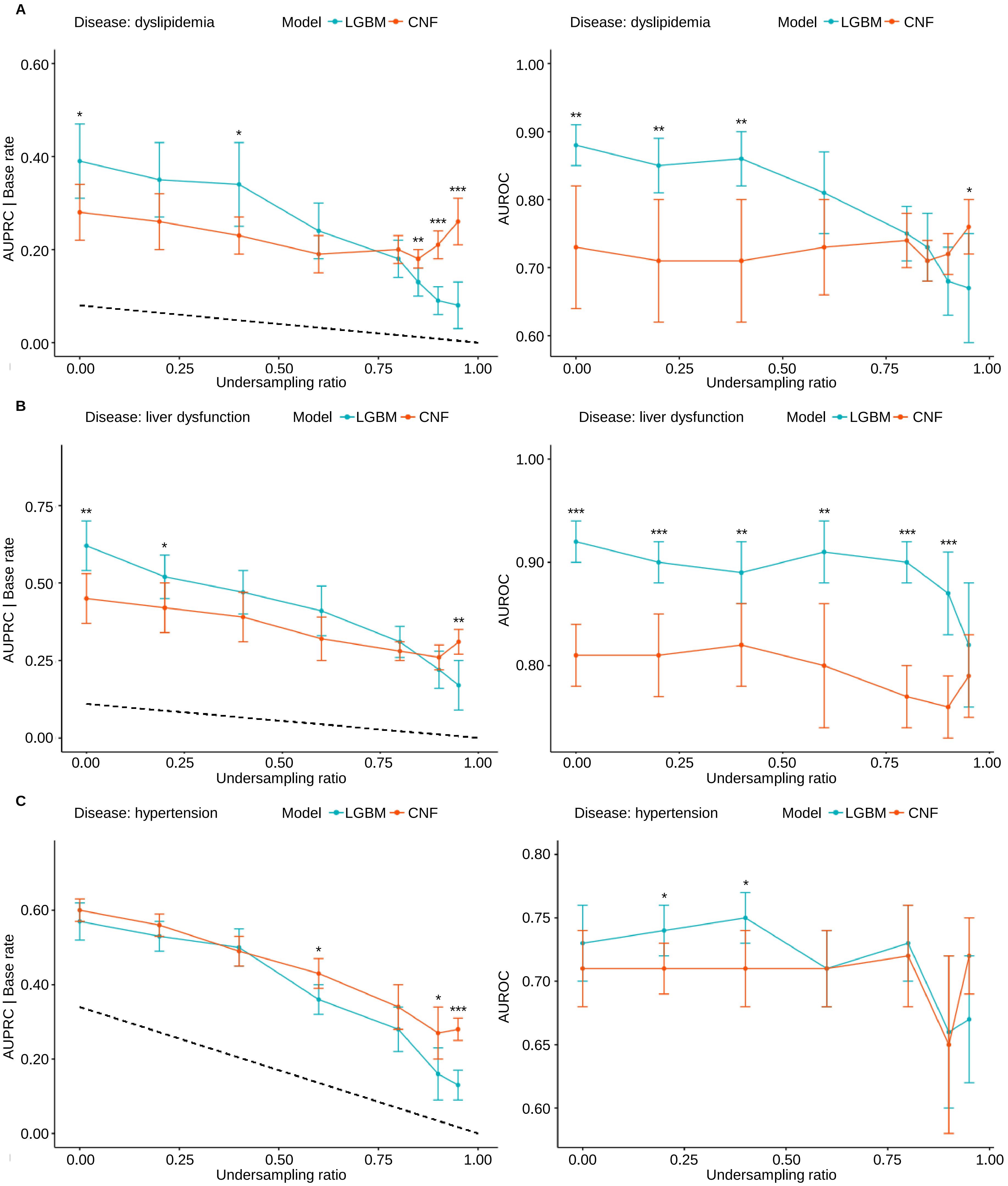

Supplementary Figure S3. The relationship between positive undersampling ratio and performance of LGBM (blue) and normalizing flow (NF, red) models on the other chronic diseases: (A) dyslipidemia, (B) liver dysfunction, and (C) hypertension. The dashed line indicates the actual base rate after adjustment of the number of positive samples. The left and right panels represent AUROC and AUPRC, respectively. The AUPRC of LGBM dropped dramatically with extreme low base rates. (A-C) Statistical analysis by Welch's t-test, *p<0.05, **p<0.01, ***p<0.001.

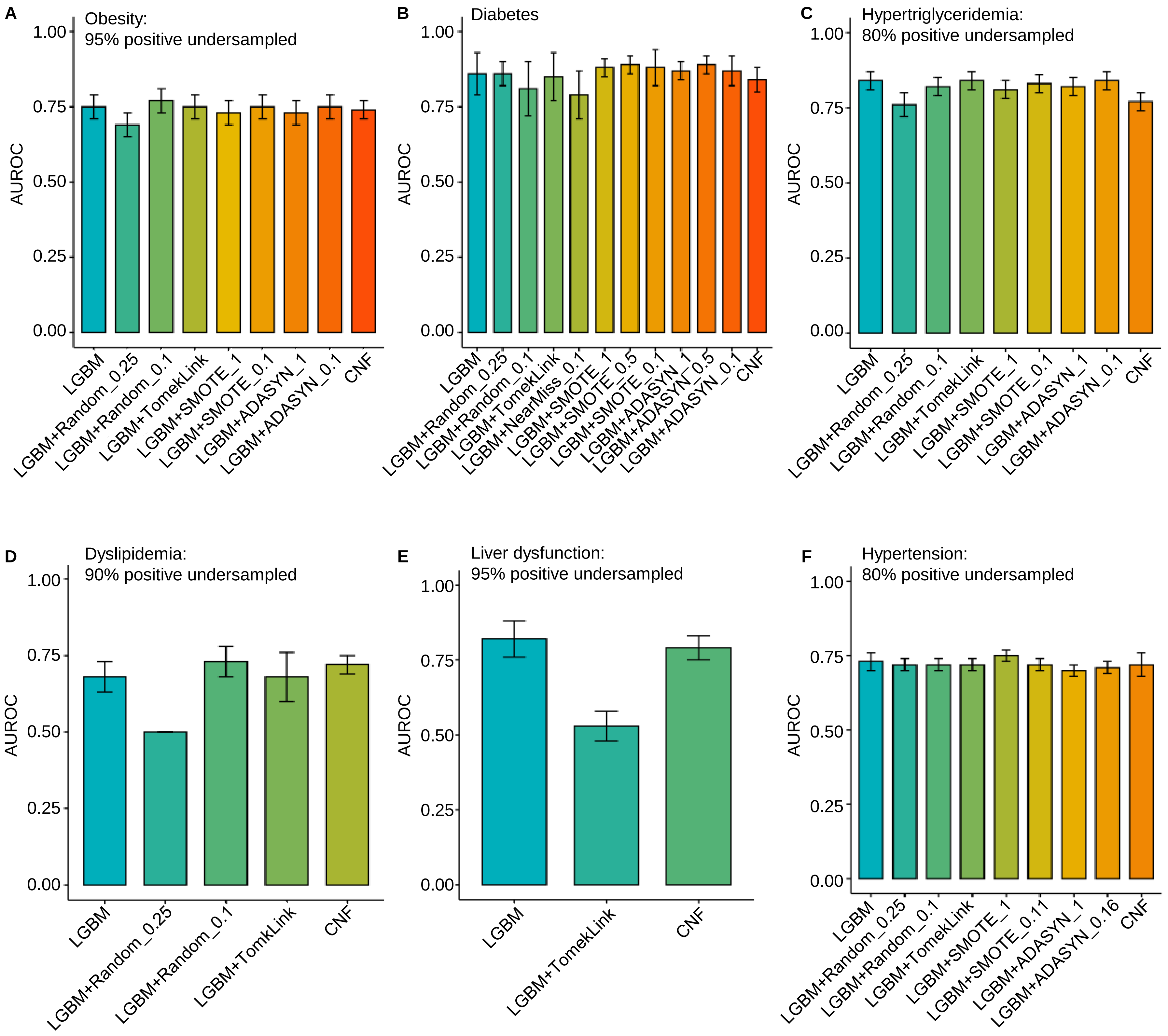

Supplementary Figure S4. The effects of positive oversampling and negative undersampling methods on AUROC. LGBM models were constructed with (positive) over- or (negative) undersampling methods for addressing class imbalance, namely Tomek Link, SMOTE, and ADASYN, for six chronic diseases: (A) Obesity: 95% undersampling, 0.02 base rate, (B) Diabetes: no undersampling, 0.02 base rate, (C) Hypertriglyceridemia: 80% undersampling, 0.02 base rate, (D) Dyslipidemia: 90% undersampling, 0.01 base rate, (E) Liver dysfunction: 95% undersampling, 0.01 base rate, and (F) Hypertension: 80% undersampling, 0.07 base rate. The dashed line indicates the actual base rate after adjustment of the number of positive samples. Positive undersampling was performed as needed to create a class imbalance situation. In the case of extreme class imbalance, (positive) oversampling or (negative) undersampling had little beneficial effect on the performance of LGBM-based classification models, whereas normalizing flow showed consistently good performance.

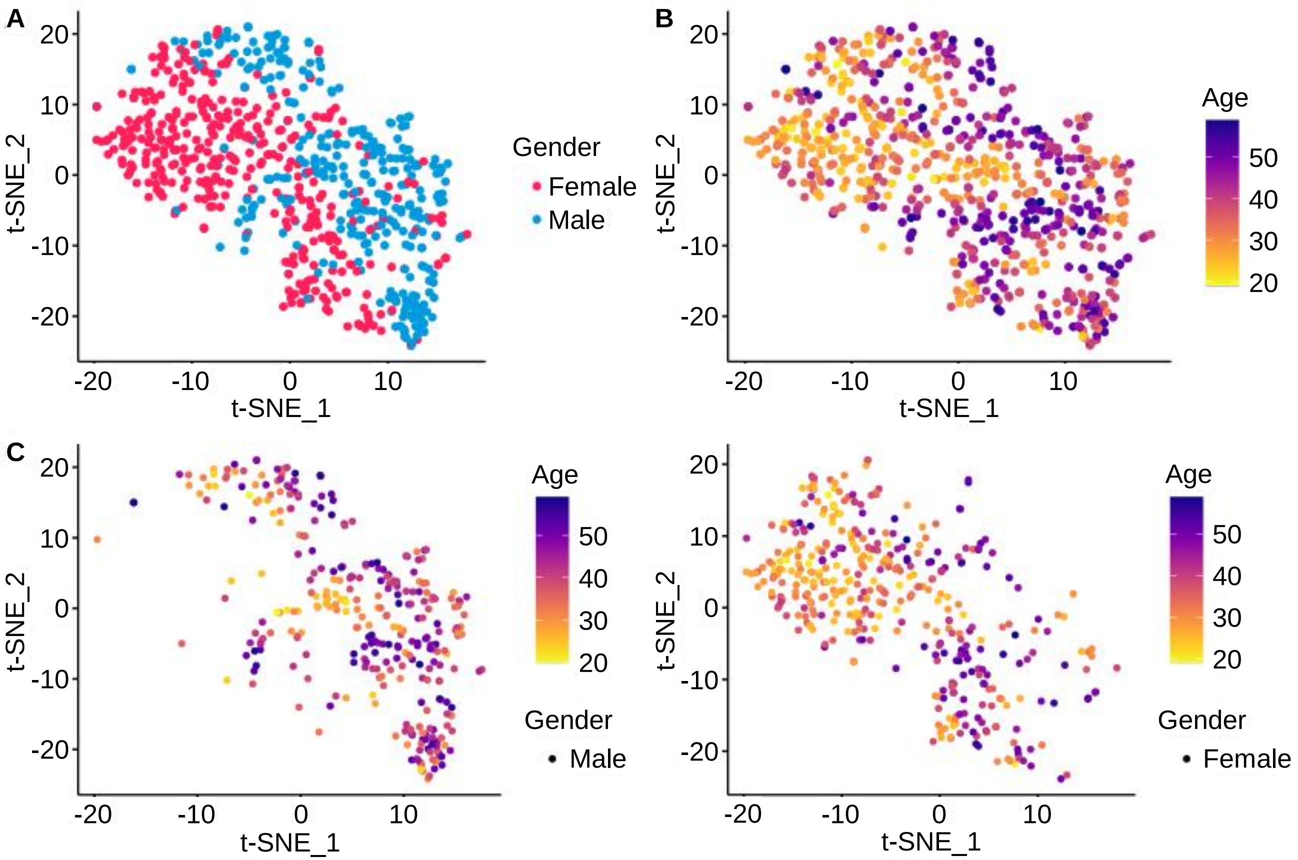

Supplementary Figure S5. (A) Dimensionality reduction to two using t-distributed stochastic neighbor embedding (t-SNE). Data points are significantly separated according to gender, which indicates a large difference in features by gender. (B) The same plot is labeled by age. A correlation is evident between age and data point position. (C) The same plot is with solely male or female participants, labeled by age. It can be seen that the position of the data point and the age have a great relationship (especially in females).
