# Supplementary material for: A machine learning approach using conditional normalizing flow to address extreme class imbalance problems in personal health records
Source: BioData Min. 2024 May 25;17:14. doi: 10.1186/s13040-024-00366-0 (PMC11127363; doi:10.1186/s13040-024-00366-0)
Supplement: Supplementary file 2 — Supplementary Material 2. [file 13040_2024_366_MOESM2_ESM.pdf]

Supplementary Table S1. Feature list of the PHR dataset before preprocessing

Feature type	Feature list	Number of features
DTC-derived genetic data	acne_result, aerobic_exercise_compatibility_result, alcohol_depend_result, alcohol_flushing_result, alcohol_meta_result, alopecia_areata_result, alopecia_M_result, appetite_result, arthritis_sensitivity_result, atopic_result, bitterness_sensitivity_result, blood_glucose_result, blood_pressure_result, BMI_result, body_fat_percentage_result, Ca_C_result, caffeine_depend_result, caffeine_meta_result, dead_skin_result, endurance_exercise_compatibility_result, Fe_C_result, freckles_result, grip_strength_result, hair_thickness_result, HDL_result, inflammatory_acne_result, insomnia_result, K_C_result, L_arginine_result, LDL_result, Mg_C_result, morning_night_person_result, motion_sickness_result, muscle_result, nicotine_depend_result, nicotine_meta_result, obesity_result, omega3_result, omega6_result, pain_sensitivity_result, pigmentation_result, psoriasis_result, recovery_after_exercise_result, satiety_result, SDMA_result, skin_aging_result, slaty_sensitivity_result, sleeping_time_result, sprint_result, strength_training_compatibility_result, sweetness_sensitivity_result, tanning_result, total_cholesterol_result, triglyceride_result, uric_acid_result, vitC_C_result, vitD_C_result, wine_acidity_result, wine_body_result, wine_flavor_result, wine_sweetness_result, wine_tannin_result, Zn_C_result	63
Medical check-up data	gender, age, height, weight, waist, bmi, left_eye, right_eye, max_bp, min_bp, hemoglobin, blood_sugar, got, gpt, gamma_gtp, cholesterol, neutral_fat, hdl, ldl, creatin, gfr	21
Survey-derived lifelog data	diet_vegis_per_month, diet_fruits_per_month, diet_seaweeds_per_month, Dscore_Hypertension, Dscore_Diabetes, Dscore_Anemia, Dscore_Dyslipidemia, Dscore_Hypertriglyceridemia, Dscore_Liver_panel, Dscore_Obesity, have_smoking, smoking_duration_all, smoking_all_count, secondary_smoking_home, secondary_smoking_home_count_per_week, secondary_smoking_duration_home, secondary_smoking_hour_home, secondary_smoking_work, secondary_smoking_work_per_week, secondary_smoking_duration_work, secondary_smoking_hour_work, drink_count_per_month, drink_amount_per_once, exercise_count_per_week, exercise_duration_hour, heavy_exercise_count_per_week, heavy_exercise_hour, medium_exercise_count_per_week, medium_exercise_hour, walk_exercise_count_per_week, walk_exercise_hour, health_condition_check, disease_exp, house_type, house_floor, house_built_year, family_number, room_number, heat_type, heat_fuel, cool_type, cook_fuel, normal_vent, normal_vent_count, normal_vent_hour, hot_vent, hot_vent_count, hot_vent_hour, freezing_vent, freezing_vent_count, freezing_vent_hour, electric_airclean_home,	139

	electric_airclean_work, electric_humidifier_home, electric_humidifier_work, electric_aircon_home, electric_aircon_work, electric_dehumid_home, electric_dehumid_work, electric_drier_home, electric_gasdrier_home, electric_gasheater_home, electric_gasheater_work, electric_oilheater_home, electric_oilheater_work, electric_fireplace_home, electric_fireplace_work, bad_airclen_working_hour, normal_airclen_working_hour, good_airclen_working_hour, kitchen_separation, use_kitchen_fan_per_ten, clean_kitchen_fan_per_month, have_molds, smell_molds, floor_material, have_rug, have_rug_carpet, have_rug_rubber, have_rug_noiseprevention, have_bed, have_curtain, have_curtain_livingroom, have_curtain_room, have_curtain_veranda, house_is_new, have_pet, pet_type_cat, pet_type_dog, pet_type_bird, have_plant, count_plant, count_big_plant, closest_road, road_lanes, road_traffic, weekday_work_hour, weekday_facility_hour, weekday_indoor_hour, weekday_outdoor_hour, weekday_walk_hour, weekday_bike_hour, weekday_car_hour, weekday_bus_hour, weekday_subway_hour, weekend_work_hour, weekend_facility_hour, weekend_indoor_hour, weekend_outdoor_hour, weekend_walk_hour, weekend_bike_hour, weekend_car_hour, weekend_bus_hour, weekend_subway_hour, multivitamin_per_week, vitaminc_per_week, vitamind_per_week, vitamine_per_week, aspirin_per_week, fe_per_week, ca_per_week, omega3_per_week, probiotics_per_week, last_school, marriage_status, average_income, economic_status, work_exp, work_title, work_start_age, last2week_symptom_decreasedintertest_in_last2weeks, last2week_symptom_depressed_in_last2weeks, last2week_symptom_sleepdisorder_in_last2weeks, last2week_symptom_tiredness_in_last2weeks, last2week_symptom_eatingdisorder_in_last2weeks, last2week_symptom_discourage_in_last2weeks, last2week_symptom_decreasedconcentration_in_last2weeks, last2week_symptom_anxious_in_last2weeks, last2week_symptom_selfharm_in_last2weeks	
Smart band-derived lifelog data	stepcount_mean_per_day, sleeptime_mean, exercise_monthly_mean_calorie, exercise_monthly_mean_count, heartrate_mean, heartrate_median	6

Supplementary Table S2. Target definition criteria

Target variable	Criteria	Category	Simple category	Unit
Obesity (BMI) [1]	<18.5	Underweight	Unaffected	kg/m^2
	18.5-22.9	Unaffected		
	23-24.9	Pre-obese		
	25-29.9	Obesity class 1	Obese	
	30-34.9	Obesity class 2		
	≥ 35	Obesity class 3		
Diabetes (Blood glucose) [2]	< 100	Unaffected	Unaffected	mg/dL
	100-125	Prediabetes		
	≥ 126	Diabetes	Diabetes	
Hypertriglyceridemia (Neutral fat) [3]	< 200	Unaffected	Unaffected	mg/dL
	200-499	High	Hypertriglyceridemia	
	≥ 500	Very high		
Dyslipidemia (HDL, LDL) [4]	HDL ≥ 40 and LDL <160	Unaffected	Unaffected	mg/dL
	HDL < 40 or LDL ≥ 160	Dyslipidemia	Dyslipidemia	
Liver dysfunction (AST, ALT) [5]	AST ≤ 40 and ALT ≤ 40	Unaffected	Unaffected	IU/L
	AST > 40 or ALT > 40	Liver dysfunction	Liver dysfunction	
Hypertension (Blood pressure / systolic, diastolic) [6]	Systolic bp < 130 and Diastolic bp < 80	Unaffected	Unaffected	mmHg
	Systolic bp ≥ 130 or Diastolic bp ≥ 80	Hypertension	Hypertension	

Supplementary Table S3. Preprocessing steps and preprocessed features

Preprocessing steps	Preprocessed features
log-transformation	got, gpt, gamma_gtp, neutral_fat, stepcount_mean_per_day, exercise_monthly_mean_calorie, count_plant
dropping sparse or unimportant features	genetic data: very sparse data distributions appeared for all variables.
	exercise_monthly_mean_count, heartrate_mean, heartrate_median, diet_vegis_per_month, diet_fruits_per_month, diet_seaweeds_per_month, Dscore_Hypertension, Dscore_Diabetes, Dscore_Anemia, Dscore_Dyslipidemia, Dscore_Hypertriglyceridemia, Dscore_Liver_panel, Dscore_Obesity, have_smoking, smoking_duration_all, smoking_all_count, secondary_smoking_home, secondary_smoking_home_count_per_week, secondary_smoking_duration_home, secondary_smoking_hour_home, secondary_smoking_work, secondary_smoking_work_per_week, secondary_smoking_duration_work, secondary_smoking_hour_work, drink_count_per_month, drink_amount_per_once, exercise_count_per_week, exercise_duration_hour, heavy_exercise_count_per_week, heavy_exercise_hour, medium_exercise_count_per_week, medium_exercise_hour, walk_exercise_count_per_week, walk_exercise_hour, health_condition_check, disease_exp, house_type, house_floor, house_built_year, family_number, room_number, heat_type, heat_fuel, cool_type, cook_fuel, normal_vent, normal_vent_count, normal_vent_hour, hot_vent, hot_vent_count, hot_vent_hour, freezing_vent, freezing_vent_count, freezing_vent_hour, electric_airclean_home, electric_airclean_work, electric_humidifier_home, electric_humidifier_work, electric_aircon_home, electric_aircon_work, electric_dehumid_home, electric_dehumid_work, electric_drier_home, electric_gasdrier_home, electric_gasheater_home, electric_gasheater_work, electric_oilheater_home, electric_oilheater_work, electric_fireplace_home, electric_fireplace_work, bad_airclen_working_hour, normal_airclen_working_hour, good_airclen_working_hour, kitchen_separation, use_kitchen_fan_per_ten, clean_kitchen_fan_per_month, have_molds, smell_molds, floor_material, have_rug, have_rug_carpet, have_rug_rubber, have_rug_noiseprevention, have_bed, have_curtain, have_curtain_livingroom, have_curtain_room, have_curtain_veranda, house_is_new, have_pet, pet_type_cat, pet_type_dog, pet_type_bird, have_plant, count_plant, count_big_plant, closest_road, road_lanes, road_traffic, weekday_work_hour, weekday_facility_hour, weekday_indoor_hour, weekday_outdoor_hour, weekday_walk_hour, weekday_bike_hour, weekday_car_hour, weekday_bus_hour, weekday_subway_hour, weekend_work_hour, weekend_facility_hour, weekend_indoor_hour, weekend_outdoor_hour, weekend_walk_hour, weekend_bike_hour, weekend_car_hour, weekend_bus_hour, weekend_subway_hour, multivitamin_per_week, vitaminc_per_week, vitamind_per_week, vitamine_per_week, aspirin_per_week, fe_per_week, ca_per_week, omega3_per_week, probiotics_per_week, last_school, marriage_status, average_income, economic_status, work_exp, work_title, work_start_age

dimensionality reduction with PCA	• ['have_smoking', 'smoking_duration_all', 'smoking_all_count'] to 'pca_smoking_all'<br>• ['secondary_smoking_home', 'secondary_smoking_home_count_per_week', 'secondary_smoking_duration_home', 'secondary_smoking_hour_home'] to 'pca_secondary_smoking_home'<br>• ['secondary_smoking_work', 'secondary_smoking_work_per_week', 'secondary_smoking_duration_work', 'secondary_smoking_hour_work'] to 'pca_secondary_smoking_work'<br>• ['last2week_symptom_decreasedintertest_in_last2weeks', 'last2week_symptom_depressed_in_last2weeks', 'last2week_symptom_sleepdisorder_in_last2weeks', 'last2week_symptom_tiredness_in_last2weeks', 'last2week_symptom_eatingdisorder_in_last2weeks', 'last2week_symptom_discourage_in_last2weeks', 'last2week_symptom_decreasedconcentration_in_last2weeks', 'last2week_symptom_anxious_in_last2weeks', 'last2week_symptom_selfharm_in_last2weeks'] to 'pca_recent_symptom_0', ‘pca_recent_symptom_1’
others	[‘left_eye’, ‘right_eye’] to ‘eye’: adopted mean value

References

1. Seo MH, Lee WY, Kim SS, Kang JH, Kang JH, Kim KK, Kim BY, Kim YH, Kim WJ, Kim EM *et al*: 2018 Korean Society for the Study of Obesity Guideline for the Management of Obesity in Korea (vol 28, pg 40, 2019). *J Obes Metab Syndr* 2019, 28(2):143-143.
2. Bansal N: Prediabetes diagnosis and treatment: A review. *World J Diabetes* 2015, 6(2):296-303.
3. National Cholesterol Education Program Expert Panel on Detection E, Treatment of High Blood Cholesterol in A: Third Report of the National Cholesterol Education Program (NCEP) Expert Panel on Detection, Evaluation, and Treatment of High Blood Cholesterol in Adults (Adult Treatment Panel III) final report. *Circulation* 2002, 106(25):3143-3421.
4. Kopin L, Lowenstein CJ: Dyslipidemia. *Annals of internal medicine* 2017, 167(11):ITC81-ITC96.
5. Siddiqui MB, Patel S, Bhati C, Reichman T, Williams K, Driscoll C, Liptrap E, Rinella ME, Sterling RK, Siddiqui MS: Range of Normal Serum Aminotransferase Levels in Liver Transplant Recipients. *Transplant Proc* 2019, 51(6):1895-1901.
6. Unger T, Borghi C, Charchar F, Khan NA, Poulter NR, Prabhakaran D, Ramirez A, Schlaich M, Stergiou GS, Tomaszewski M *et al*: 2020 International Society of Hypertension Global Hypertension Practice Guidelines. *Hypertension* 2020, 75(6):1334-1357.
